# The association of *Neoehrlichia mikurensis* infection with polymyalgia rheumatica

**DOI:** 10.1007/s15010-026-02857-0

**Published:** 2026-07-10

**Authors:** Christine Lingblom, Sofia Sundvall, Kerstin Andersson, Alaitz Aranburu, Kristina Bengtsson Boström, Per Hjerpe, Karin Mossberg, Pär-Daniel Sundvall, Christine Wennerås

**Affiliations:** 1 Department of Clinical Microbiology, Sahlgrenska University Hospital, Gothenburg, Sweden; 2 Department of Infectious Diseases, the Sahlgrenska Academy, University of Gothenburg, Gothenburg, Sweden; 3 Research, Education, Development and Innovation Primary Health Care, Region Västra Götaland, Gothenburg, Sweden; 4 Department of Rheumatology and Inflammation Research, Institute of Medicine, the Sahlgrenska Academy, University of Gothenburg, Gothenburg, Sweden; 5 General Practice/Family Medicine, School of Public Health and Community Medicine, Institute of Medicine, Sahlgrenska Academy, University of Gothenburg, Gothenburg, Sweden; 6 Regionhälsan R&D Centre, Skaraborg Primary Care, Skövde, Sweden

**Keywords:** *Neoehrlichia mikurensis*, Polymyalgia rheumatica, Angiopoietin-1, Insulin-like growth factor-binding protein 4, CystatinC, Interleukin-1ß, Anti-endothelial cell antibodies

## Abstract

*Neoehrlichia mikurensis* is a tick-borne bacterium that can give rise to latent infections of vascular endothelium and PMR-like symptoms. PMR is an autoinflammatory or autoimmune condition of unknown cause that has been linked to infectious agents. The objective of this study was to investigate a possible association of PMR with *N. mikurensis* infection. Patients with PMR (*n* = 142) and matched control subjects (*n* = 56) from seven primary health care centers in West Sweden were tested for infection with *N. mikurensis* by PCR and serology. Patient-reported data regarding 31 PMR symptoms, tick exposure, past and current co-morbidities were registered. Anti-endothelial cell antibodies (AECA) and 38 inflammatory markers were measured in serum. The data was analyzed by multivariate methods of pattern recognition. There was no difference in the prevalence of infection with *N. mikurensis* between the PMR patients (5.6%) and the control subjects (5.4%). PMR patients infected with *N. mikurensis* differed from the non-infected PMR patients by their raised levels of IgM AECA and of interleukin-1β, and lower levels of insulin-like growth factor-binding protein 4, cystatin C and angiopoietin-1. Furthermore, there was a correlation between IgM antibodies to *N. mikurensis* and IgM AECA (*r* = 0.50, *P* < 0.0001). To conclude, PMR patients infected with *N. mikurensis* had vascular autoantibodies and a distinct inflammatory profile but whether the infection had triggered or modulated the development of PMR could not be established in the present study.

**Supplementary Information:**

The online version contains supplementary material available at https://doi.org/10.1007/s15010-026-02857-0.

## Introduction

*Neoehrlichia (N.) mikurensis* is an intracellular bacterial species that is transmitted via tick bites and causes the infectious disease neoehrlichiosis [1]. This zoonotic infection is widely spread among ticks and wild rodents in Europe and northern Asia [2]. *N. mikurensis* can only be diagnosed by PCR of blood samples since it does not grow outside of cells and consequently is missed by blood culture, leading to diagnostic delays and missed diagnoses [2]. We have evidence that *N. mikurensis* infects vascular endothelial cells, which probably explains the vascular events that frequently accompany the infection [3]. Importantly, many cases of infection are latent and may reactivate once immune defenses are compromised [4]. Neoehrlichiosis is an infection of older adults – there are no reports of pediatric infection and the median age of the largest cohort of patients described up to date, composed of 102 Swedish patients, was 62 [5]. The clinical picture of neoehrlichiosis varies depending on immune status. Immunocompromised patients often have fever with chills, fatigue and vascular events of the venous circulation, whereas neoehrlichiosis in immunocompetent individuals can range from asymptomatic infection to severe vasculitis of medium- to large-sized arteries mimicking systemic rheumatic diseases [3, 5]. Some patients with neoehrlichiosis have reported PMR-like symptoms such as fatigue, stiff legs and muscular pain often located to the neck [3, 5].

Polymyalgia rheumatica (PMR) is an inflammatory rheumatic disease that mainly afflicts people above the age of 50 and is twice as common in women [6]. The highest incidence of PMR is found in Scandinavia and in people of northern European descent [6]. Typical symptoms are stiffness and muscular pain of the neck, shoulders, lower back, and thighs that may be accompanied by low-grade fever and fatigue. Immune cell infiltration of muscles, periarticular regions and blood vessels have been observed in PMR [7]. Up to 20% of newly diagnosed PMR patients also have giant cell arteritis (GCA), an inflammatory condition affecting the large arteries, and an additional 23–29% may have subclinical GCA that can be detected by imaging techniques [8].

The immunopathogenesis of PMR is not fully understood. It is unclear whether it is primarily an autoimmune condition, or an autoinflammatory one, or both [9]. No autoantibodies specific for PMR have been identified, and corticosteroids are currently the mainstay of treatment [6, 9]. Although the cause of PMR is unknown, a multifactorial etiology has been suggested [9]. Several infectious candidates have been proposed as possible triggers of PMR. A rise in the incidence of PMR has been observed in connection to epidemics of *Mycoplasma pneumoniae*, *Chlamydia pneumoniae* and Parvovirus B19 in Denmark [10]. Further, there have been case reports describing the development of PMR after, or concurrent with infection due to influenza virus B [11], varicella zoster virus reactivation [12], and *Babesia microti *[13].

The unknown and purportedly heterogeneous etiology of PMR, the tropism of *N. mikurensis* for arterial blood vessels, and the PMR-like clinical picture of some cases of neoehrlichiosis led us to investigate the potential association of this emerging pathogen with PMR. We hypothesized that *N. mikurensis* infection might trigger the development of PMR in some cases, and/or that there might be cases of *N. mikurensis* infection misdiagnosed as PMR. To resolve these questions, a cohort of patients with active PMR was recruited from seven primary care centers in the Västra Götaland Region of Sweden. The incidence of *N. mikurensis* infection was determined in this cohort and compared to a cohort of age-matched subjects without PMR residing in the same geographic area. We also investigated the immune profiles and clinical pictures of PMR patients with and without *N. mikurensis* infection to determine if PMR associated with *N. mikurensis* infection differs from “ordinary” PMR.

## Materials and methods

### Study design

This multicenter study encompassed seven primary care clinics in the Västra Götaland Region of Sweden: the Närhälsan vårdcentral of Norrmalm, Billingen, Herrestad, Dagson, Sandared, Ågårdskogen and Bollebygd (Supplementary Fig. 1). All patients with a current diagnosis of PMR were offered participation in the study. The study nurse (SS) interviewed all recruited patients by telephone employing a questionnaire specifically designed for the study to cover demographic factors, co-morbidities, duration and symptoms of PMR, symptoms typical of neoehrlichiosis, and current medication including corticosteroids (Supplementary Table 1). The PMR patients were also asked to suggest a spouse/partner/relative/friend/neighbor who might want to participate in the study as a control subject. The control subjects had to live in the same geographic area, be of a similar age (± 10 yrs), subjectively healthy, and with no history of PMR. The control subjects filled in a written questionnaire with health-related questions. Patients and controls alike answered a questionnaire covering history of tick exposure and tick-borne diseases. All data were stored in an Electronic Case Report Form (Research Electronic Data Capture RedCap, Gothia Forum, Gothenburg, Sweden). Study patients and control subjects donated blood (serum and EDTA-blood) to diagnose *N. mikurensis* by PCR and for measurement of inflammatory markers, antibodies to *N. mikurensis* and to *Borrelia burgdorferi* sensu lato, and endothelial autoantibodies. ESR, CRP, WBC, and acute phase reactants were retrieved from the patients’ medical charts to assess the inflammatory profile of PMR at the time of diagnosis. Study subjects who tested positive for *N. mikurensis* were recommended eradication therapy with oral doxycycline 100 mg twice daily for 3 weeks. Follow-up after treatment consisted of control PCR for *N. mikurensis*. Clinical symptom follow-up was not done because it was not included in the ethical approval for the study.

### Ethical approval

The study was approved by the Swedish Ethical Review Authority (2021 − 00903). All study participants provided oral and written informed consent.

### *N. mikurensis* PCR

Fresh EDTA-blood (1 mL) was used for the specific RT-PCR [14], which was performed at the Department of Clinical Microbiology, Sahlgrenska University Hospital, Gothenburg, the National Reference Laboratory for neoehrlichiosis in Sweden.

### *N. mikurensis* serology

Thawed serum was tested for IgM and IgG antibodies to *N. mikurensis* using a recently developed in-house ELISA based on an outer membrane protein (P44/Msp2) of *N. mikurensis* [15]. Antibody levels are expressed as the optical-density (OD) values for serum diluted 1:100.

### Borrelia serology

The chemiluminescence immunoassays Liaison Borrelia IgM and IgG (DiaSorin, Saluggia, Italy) were used to measure IgM and IgG antibodies to *Borrelia burgdorferi* sensu lato in serum. Antibody concentrations were expressed as Arbitrary Units (AU)/mL. The cut-off levels for the assays were IgM < 22 and IgG < 15 AU/mL.

### Inflammatory markers

Thawed serum was analyzed using the Legendplex “Essential immune response”, “Anti-virus response”, “Angiogenesis” and “Human vascular inflammation” multiplex kits (Biolegend) to measure a total of 38 cytokines and growth factors listed in Supplementary Table 2.

### Anti-endothelial cell antibodies (AECA)

An in-house ELISA for the detection of AECA was developed. Antigen preparations of cytosolic and membrane proteins were made from primary human pulmonary artery endothelial cells (PromoCell) grown to confluence in 75 cm^2^ flasks (Greiner 658175) in Endothelial Cell Growth Medium 2 (PromoCell). The cells were processed using the Mem-PER Plus Membrane Protein Extraction Kit protocol for adherent mammalian cells (ThermoFisher Scientific). Cells were permeabilized with a mild detergent to release the cytosolic fraction of proteins and treated with a second detergent to solubilize and isolate the membrane fraction of proteins. Protein concentrations were determined with the BCA Protein Assay kit (Pierce) using a ND-1000 NanoDrop Spectrophotometer. Protease Inhibitor Cocktail (Sigma-Aldrich) was added to the protein fractions at 1% (vol/vol) and stored at -140 °C until use. Half-area medium-binding 96-well plates (Corning) were coated with the membrane or cytosolic protein fractions diluted in PBS to 3 µg/mL, + 4 °C overnight. Blank wells were coated with PBS. From here on, all steps were performed at RT. Plates were washed twice with 0.05% PBS-Tween20 (PBST) (Sigma-Aldrich) and blocked with 1% BSA-PBS (Sigma-Aldrich) for 1 h. During the blocking step, thawed serum samples were pre-incubated with 10% fetal bovine serum for 15–30 min to remove antibodies against bovine serum proteins. Next, plates were washed once with PBST and serum samples diluted 1:50 in 1% BSA-PBS were added in duplicate and incubated for 2 h. Plates were washed thrice with PBST before addition of goat anti-human IgG-HRP or goat anti-human IgM-HRP (Invitrogen), both diluted to 0.5 µg/mL in 1% BSA-PBS and incubated for 2 h. Plates were then washed thrice with PBST and incubated with TMB substrate (BD Biosciences) for 10–15 min for IgG and 25–30 min for IgM, before stopping the reaction by the addition of 1M H_2_SO_4_. Optical density (OD) was measured at 450 nm in an ELISA-reader (Molecular Devices SpectraMax ABS Plus with SoftMax Pro 7.3.1 Software). OD-values for the uncoated wells were deducted from the mean value of the antigen-coated wells.

### Data analysis and statistics

Mann-Whitney non-paired test and Fisher’s exact test were used to compare two groups, and correlations between datasets were assessed by the Spearman’s rank correlation test using GraphPad PRISM 9.2.0 software (GraphPad). P-values < 0.05 were considered statistically significant. The multivariate method Projections to Latent Structures by means of Partial Least Squares-Discriminant Analysis (PLS-DA) was performed using the SIMCA-P (version 15.0.2) statistical package (MKS Data Analytics Solutions, Malmö, Sweden). Variable Importance in the Projection (VIP) scores were calculated. VIP is a measure derived from the projection of the Partial Least Squares Discriminant Analysis (PLS-DA) model, which estimates the contribution of each variable to the model. We chose a cut-off of 1 as variables with VIP scores > 1 are generally considered significant. However, setting 1 as a threshold is only a general guideline and should be verified with univariate analysis [16]. The quality of the multivariate models was determined by their explanatory power (R2Y) and robustness (Q2Y).

## Results

### Study population

A total of 142 patients with PMR and 56 control subjects were recruited to the study. The patients and controls were well-matched regarding age, sex, and geography (Table 1). The PMR patients had a median disease duration of 5 years and more than 80% had ongoing low-dose corticosteroid therapy (Table 1).

**Table 1:** Clinical characteristics of the study participants

Characteristics	PMR patients (*n* = 142)	Control subjects (*n* = 56)	*P*-value
Age, years, median (range)	75 (49–92)	72 (53–88)	NS
Female sex, n/N (%)	85/142 (60%)	33/56 (59%)	NS
Duration of PMR, years, median (range)	5 (1–7)	NA	NA
Current prednisolone treatment, n/N (%)	116/142 (82%)	1/56 (1.8%)	< 0.001*
PCR positivity for *N. mikurensis*, n/N (%)	8/142 (5.6%)	3/56 (5.4%)	NS
*Borrelia* IgG antibodies, n/N (%)	51/142 (36%)	15/56 (27%)	NS
*Borrelia* IgM antibodies, n/N (%)	16/142 (11%)	7/56 (12%)	NS

*NA* not applicable. *NS* not statistically significant. *Fisher’s exact test

### Prevalence of *N. mikurensis* infection

There was no difference in the prevalence of current (PCR-positive) infection with *N. mikurensis* between the PMR patients (5.6%) and the control subjects (5.4%), Table 1. All the infected individuals appeared to have asymptomatic infection as none of them had fever or any other overt symptoms of infection. The degree of self-reported tick exposure was also very similar between the infected and non-infected PMR patients, as were the rates of seroconversion to Borrelia (Tables 1 and 2).

**Table 2:** Prevalence of tick exposure

Tick exposure	PMR patients Infected (*n* = 8)	PMR patients Non-infected (*n* = 134)	Healthy controls Infected (*n* = 3)	Healthy controls Non-infected (*n* = 53)
Heavy	4/8 (50%)	62/134 (46%)	1/3 (33%)	30/53 (57%)
Medium	3/8 (38%)	41/134 (31%)	1/3 (33%)	14/53 (26%)
None reported	1/8 (12%)	23/134 (17%)	1/3 (33%)	10/53 (19%)

Heavy exposure, several times per year. Medium exposure, once per year

### Seroconversion to *N. mikurensis*

**Fig. 1:**
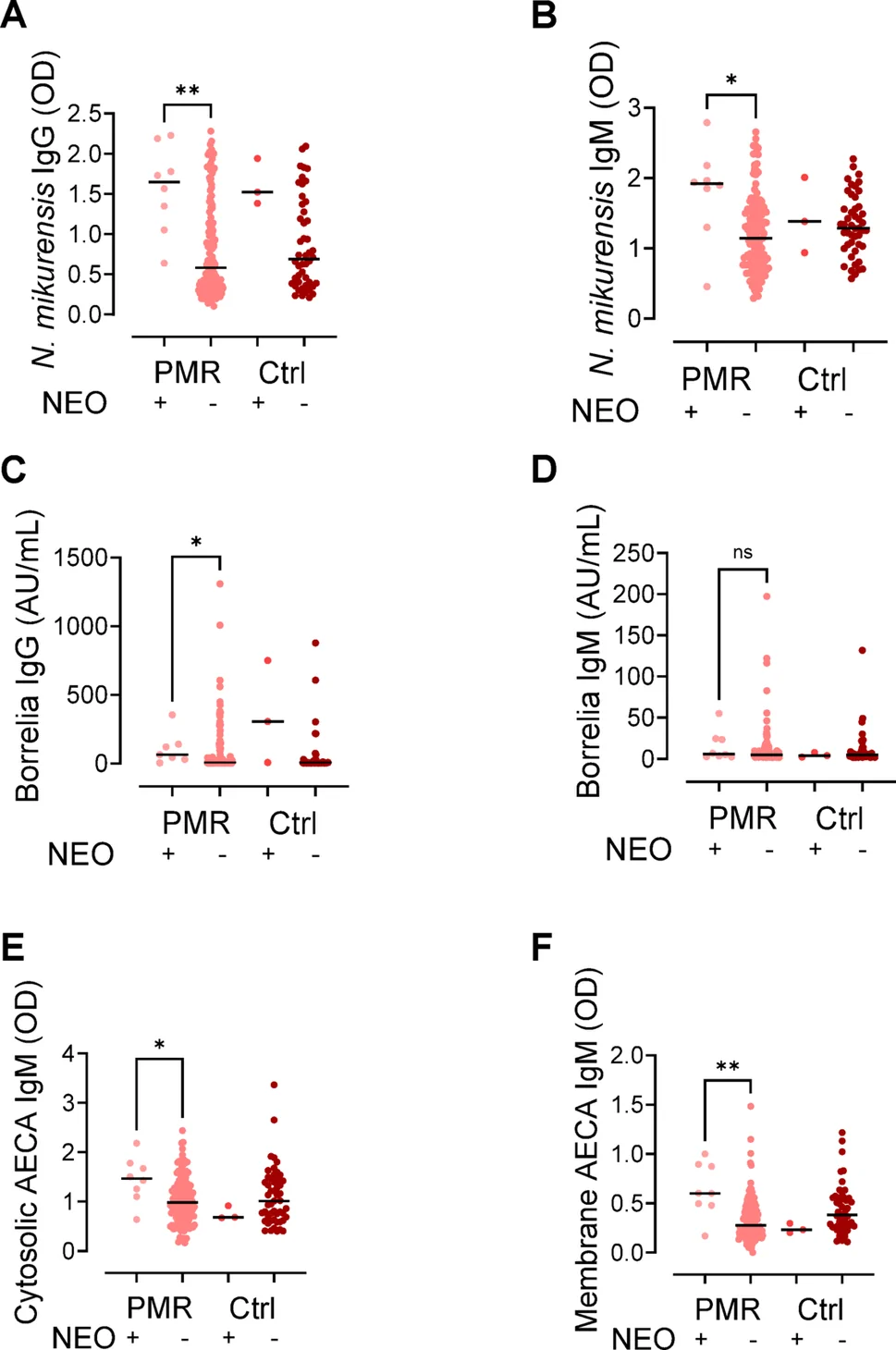
The levels of IgG (**A**) and IgM (**B**) antibodies to *N. mikurensis;* IgG (**C**) and IgM (**D**) antibodies to *B. burgdorferi;* and IgM AECA to cytosolic (**E**) and membrane (**F**) proteins of arterial endothelial cells in serum samples of PMR patients infected with *N. mikurensis* (PMR+, *n* = 8), PMR patients not infected with *N. mikurensis* (PMR-, *n* = 134), healthy controls infected with *N. mikurensis* (Ctrl+, *n* = 3) and healthy controls not infected with *N. mikurensis* (Ctrl-, *n* = 53). AECA, anti-endothelial cell antibodies. Mann Whitney U test and Spearman’s rank correlation tests with *P* < 0.05

Next, all patients were tested for antibodies to *N. mikurensis* in order not to miss patients with past exposure to *N. mikurensis* among those who tested negative for *N. mikurensis* by PCR. Although there were individuals in the group of PCR-negative PMR patients with IgM and IgG responses to *N. mikurensis* of the same magnitude as the PCR-positive PMR patients (Fig. 1A, B), at a group level, the median antibody levels were very similar between the PMR patients and the control subjects who were PCR-negative (Fig. 1A, B). As expected, the levels of IgM and IgG to *N. mikurensis* were significantly higher in the group of PCR-positive PMR patients compared with the PCR-negative PMR patients (Fig. 1A, B). We also measured the prevalence of Borrelia antibodies as a proxy of past tick bites and found statistically higher concentrations of IgG but not of IgM antibodies to Borrelia among the PCR-positive PMR patients compared to the PCR-negative ones (Fig. 1C, D). To summarize, there was no difference in the prevalence of antibodies to *N. mikurensis*, as a proxy measure of past infection with *N. mikurensis*, between the groups of PCR-negative PMR patients and control subjects, respectively.

### Relationship of antibodies to *N. mikurensis* with endothelial autoantibodies

**Fig. 2:**
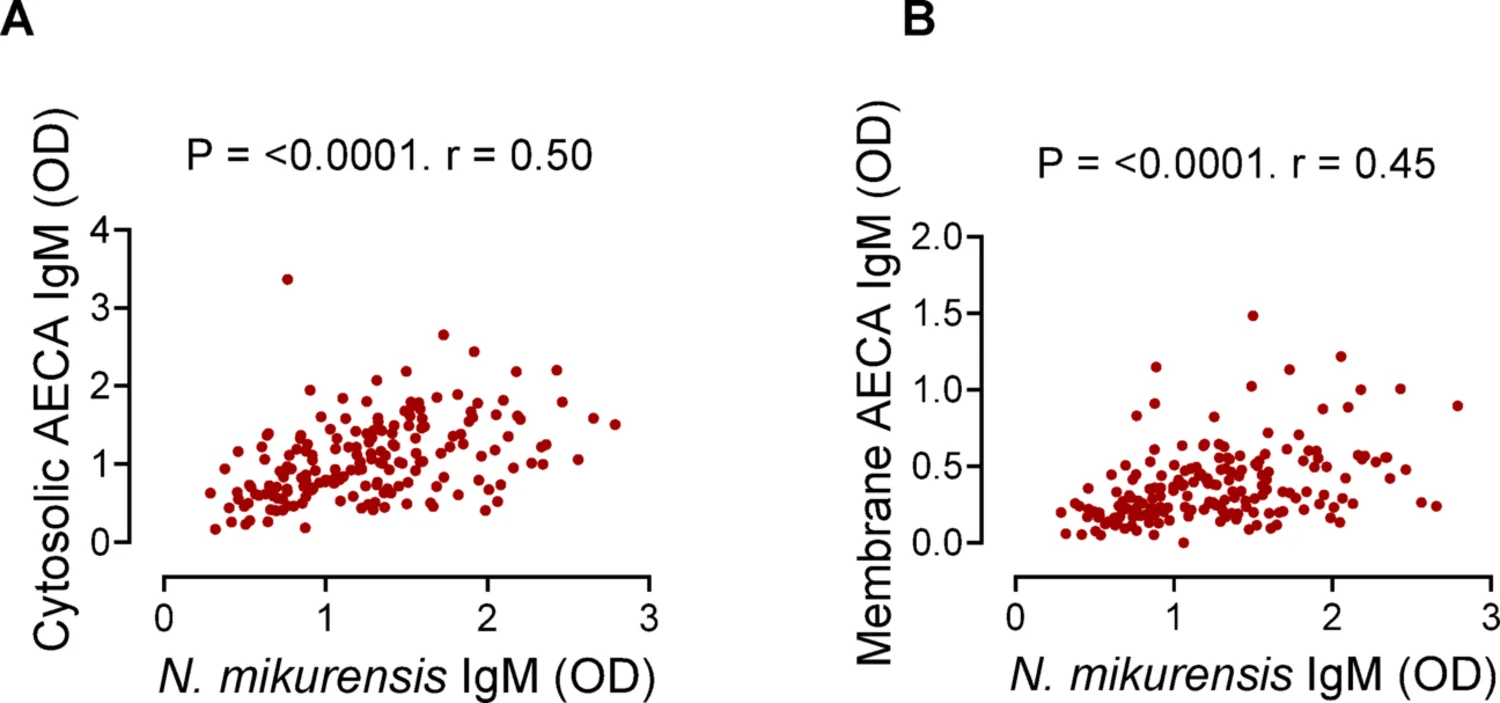
Correlations between serum levels of IgM antibodies to *N. mikurensis* and IgM AECA to cytosolic (**A**) and membrane (**B**) proteins of arterial endothelial cells. AECA, anti-endothelial cell antibodies. Spearman’s rank correlation test, *P* < 0.05

Although none of the PMR patients were diagnosed with Giant cell arteritis (GCA) we reasoned there might be patients with undiagnosed vasculitis in view of the published estimates of up to every third patient with PMR having subclinical GCA [8]. This is why we chose to study anti-endothelial cell antibodies (AECA). We opted to develop an in-house ELISA based on primary human arterial endothelial cells and not on the more frequently used human umbilical vein endothelial cells (HUVEC) since we reasoned that HUVEC is a very specialized and distinct type of endothelium that may not be relevant for infection with *N. mikurensis*. PMR patients with *N. mikurensis* infection had higher levels of IgM autoantibodies to cytosolic as well as to membrane endothelial antigens than did the non-infected PMR patients (Fig. 1E, F). No such pattern was seen for the three control subjects with *N. mikurensis* infection, who had lower than average levels of AECA, even lower than the non-infected controls (Fig. 1E, F). There was no difference between the infected and non-infected PMR patients regarding the levels of IgG AECA (data not shown). Lastly, there was a medium strong correlation (*r* = 0.45–0.50, *P* < 0.0001) between the levels of IgM antibodies to *N. mikurensis* and IgM AECA to the cytosolic and membrane fractions of human arterial cells (Fig. 2A, B). In contrast, a weak correlation was seen for IgM antibodies to Borrelia and IgM AECA to the same endothelial cell fractions (*r* = 0.33 and 0.29, respectively, both *P* < 0.0001).

### Inflammatory profile

**Fig. 3:**
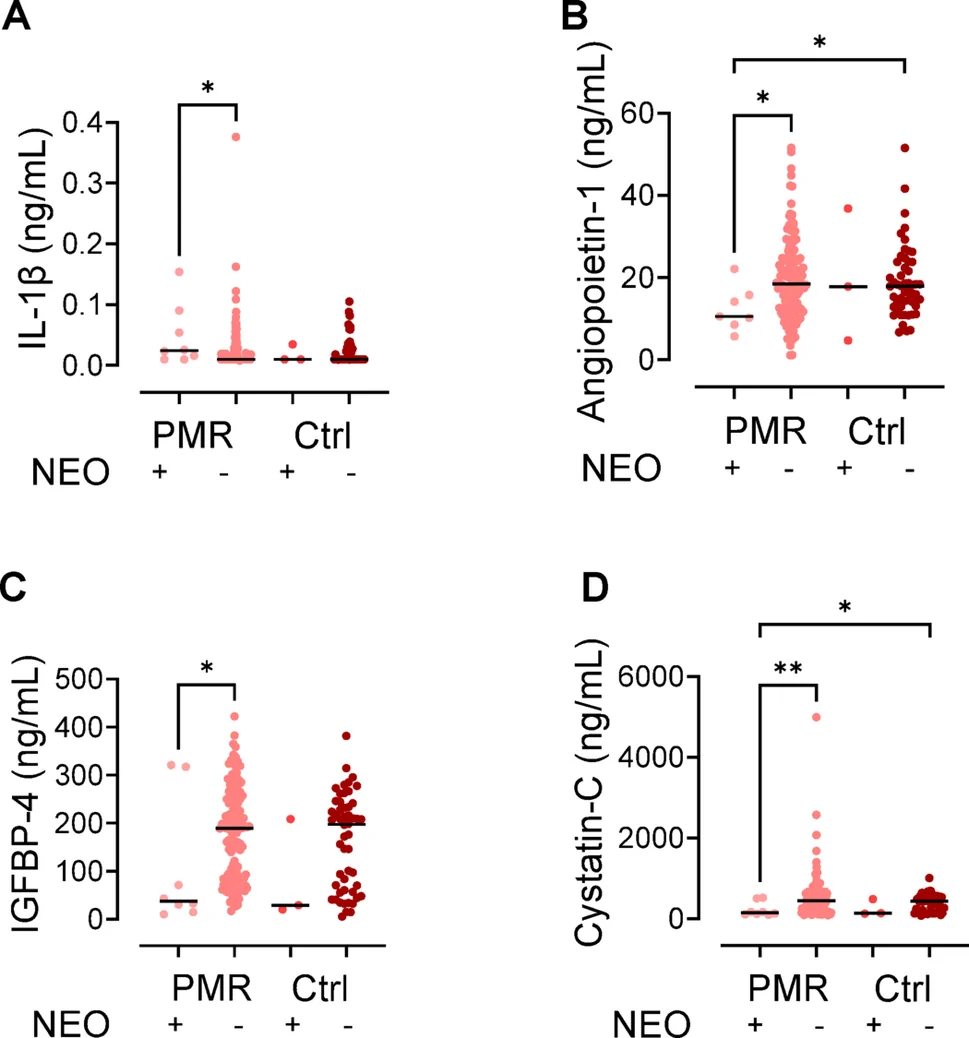
The concentrations of cytokines determined by multiplex analyses of serum samples of PMR patients infected with *N. mikurensis* (PMR+, *n* = 8), PMR patients not infected with *N. mikurensis* (PMR-, *n* = 134), healthy controls infected with *N. mikurensis* (Ctrl+, *n* = 3) and healthy controls not infected with *N. mikurensis* (Ctrl-, *n* = 53). (**A**) interleukin-1β (IL-1β), (**B**) angiopoietin-1, (**C**) insulin-like growth factor binding protein-4 (IGFBP-4) and **(D)** cystatin-C. Mann-Whitney test, *P* < 0.05

We measured the concentrations of 38 cytokines and growth factors in the serum of the infected and non-infected PMR and control patients. The analytes were selected based on their importance for vascular inflammation and immunity to intracellular pathogens such as *N. mikurensis*. The *N. mikurensis*-infected PMR patients had higher concentrations of interleukin-1β (IL-1β) than the non-infected PMR patients (Fig. 3A), and conversely, lower levels of angiopoietin-1, insulin-like growth factor-binding protein 4 (IGFB-4) and cystatin C than the non-infected PMR patients (Fig. 3B–D). It should be emphasized that most of the PMR patients were taking a low dose of oral prednisone. However, a similar depressed response pattern for IGFB-4 and cystatin C was noted for the infected versus non-infected control subjects, although there were too few infected control subjects (*n* = 3) for statistical comparisons (Fig. 3C, D).

### AECA and cytokine profiles differentiate *N. mikurensis*-infected PMR patients from non-infected PMR patients

**Fig. 4:**
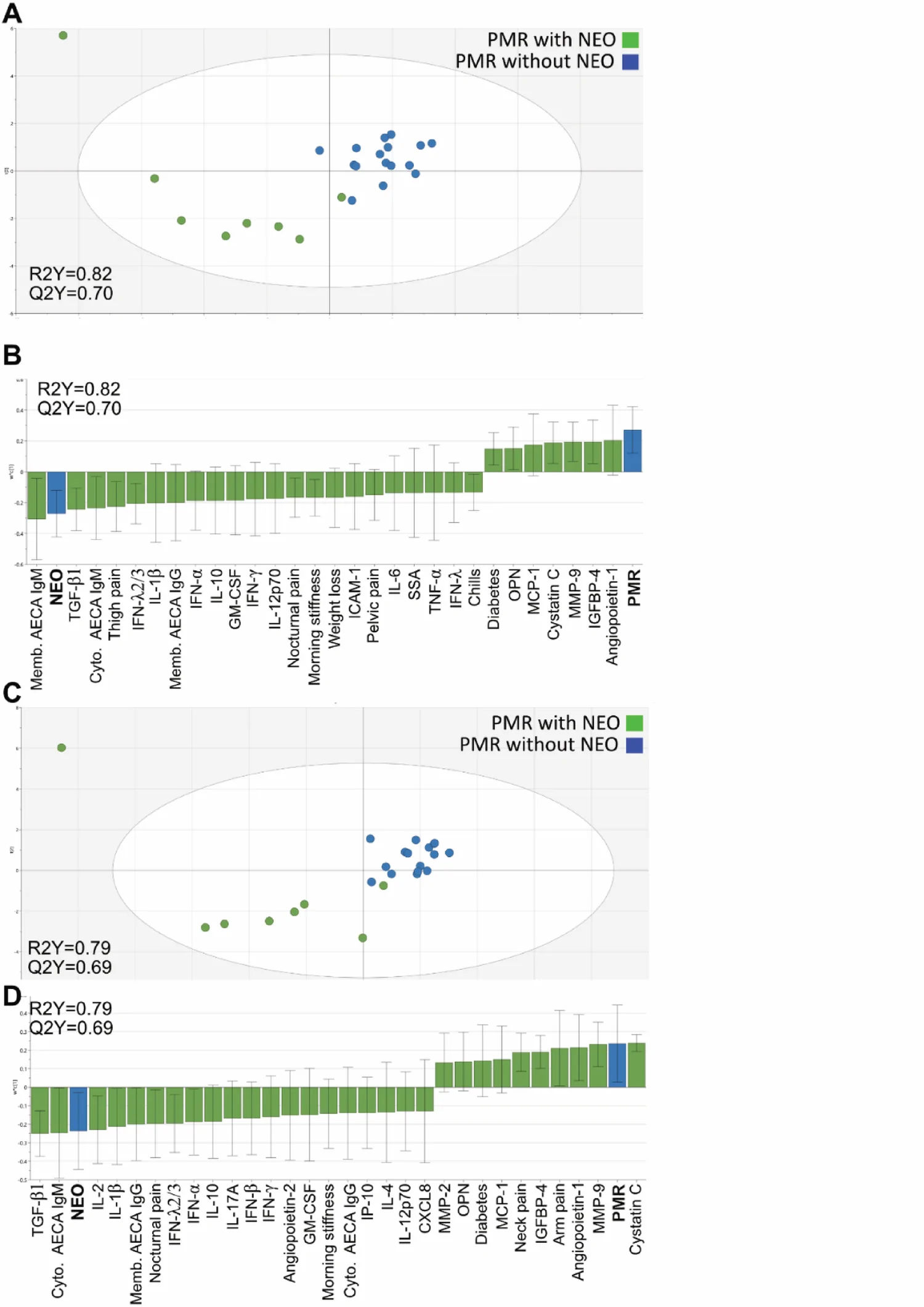
Multivariate models were constructed using Partial Least Squares-Discriminant Analysis (PLS-DA) to evaluate if inflammatory markers and clinical data could separate infected from non-infected PMR patients. (**A**) PLS-DA cluster plot showing individual patients infected with *N. mikurensis* in green (NEO; *n* = 8) and non-infected PMR in blue (PMR, *n* = 16). (**B**) Loading plot with jackknife confidence intervals for the variables with highest impact on “model A”. The markers closely positioned to the respective patient categories (NEO, infected PMR patient category; PMR, non-infected patient category) are positively associated with the patient category in question. (**C**) A second model was constructed using the same group of infected PMR patients as in A, but with a new set of controls (*n* = 16). The original set of controls in A had an average age of 74 (range 50–91) almost identical to the controls in C with an average age of 76 (65–91). (**D**). Jack-knife loading plots depicting the most discriminatory variables for model C. The generated models A and C achieved strong explanatory powers of 82% (R²Y = 0.82) and 79% (R²Y = 0.79), and stabilities of 70% (Q²Y = 0.70) and 69% (Q²Y = 0.69), respectively. Parameters associated with *N. mikurensis*-infected PMR patients are shown on the left, while those associated with non-infected PMR patients are shown on the right

Lastly, we constructed multivariate models (PLS-DA) to evaluate if it was possible to separate PMR patients infected with *N. mikurensis* from non-infected PMR patients based on the clinical data and inflammatory parameters, i.e., a total of 38 cytokines, 31 symptoms, IgM and IgG AECA, and demographic data. A first model was constructed composed of the PMR patients infected with *N. mikurensis* (*n* = 8) and two non-infected PMR patients per infected PMR patient (*n* = 16) that were matched for age, sex and geographic location. The model provided a distinct separation between the infected and non-infected patients with virtually no overlap (Fig. 4A) and a high explanatory power (82%) and good robustness (Q²Y = 0.70). The variables of importance for the separation of the infected from the non-infected PMR patients are presented in Fig. 4B. Parameters associated with *N. mikurensis*-infected PMR patients are shown on the left, while those associated with non-infected PMR patients are shown on the right. PMR patients infected with *N. mikurensis* had higher IgM AECA to membrane and cytosolic proteins and more elevated concentrations of the cytokines TGF-β1, IFN-l2/3 and IL-1β (Fig. 3B). In contrast, the matched non-infected PMR patients had higher levels of the cytokines angiopoietin-1, IGFBP-4, MMP-9, and cystatin C, Fig. 4B. Thigh pain occurred more often among the infected PMR patients than in the non-infected group of PMR patients (Fig. 4B). We checked for the risk of this model being overfit to the selected 16 non-infected PMR patients by building a second model composed of the same 8 infected PMR patients but a new set of 16 non-infected PMR patients matched for sex and age (Fig. 4C, D). This second model also provided very good discriminatory power (79%) between infected and non-infected PMR patients (Fig. 4C) with a similar pattern of discriminatory variables as the first model with regards to cytokines and AECA but differences with respect to symptoms (Fig. 4D). Arm and neck pain were relatively more frequent in the second model’s group of control subjects compared to the infected group that had relatively more nocturnal pain (Fig. 4D). To conclude, cytokine patterns and AECA were unchanging discriminatory factors to segregate infected from non-infected PMR patients in the multivariate models. In contrast, although symptoms lent stability to the models they varied between the models. In fact, symptoms and clinical signs failed to segregate infected from non-infected patients in the entire cohort of PMR patients (Table 3).

**Table 3:** Prevalence of symptoms and clinical signs among the PMR patients based on infection status with *N. mikurensis*

Symptoms	Infected *n* = 8	Non-infected *n* = 134	*P*-value
Arm pain, n/N (%)	5/8 (63%)	108/134 (81%)	NS
Back pain	4/8 (50%)	62/134 (46%)	NS
Chills	4/8 (50%)	39/134 (29%)	NS
Depression	2/8 (25%)	60/134 (45%)	NS
Fatigue	4/8 (50%)	95/134 (71%)	NS
Fever	3/8 (38%)	31/134 (23%)	NS
Headache	1/8 (12%)	36/134 (27%)	NS
High blood pressure	6/8 (75%)	88/134 (66%)	NS
Morning stiffness	8/8 (100%)	110/134 (82%)	NS
Movement pain	3/8 (38%)	42/134 (31%)	NS
Neck pain	1/8 (12%)	77/134 (58%)	0.023*
Nocturnal pain	8/8 (100%)	86/134 (64%)	NS
Pain responsive to analgesics medication	6/8 (75%)	94/134 (70%)	NS
Pelvic pain	7/8 (88%)	83/134 (62%)	NS
Rash	2/8 (25%)	24/134 (18%)	NS
Poor appetite	1/8 (12%)	29/134 (22%)	NS
Shoulder pain	4/8 (50%)	106/134 (7%)	0.077*
Stiffness	8/8 (100%)	112/134 (84%)	NS
Thigh pain	8/8 (100%)	88/134 (66%)	0.054*
Weight loss	3/8 (38%)	48/134 (36%)	NS

*NS* not statistically significant. *Fisher’s exact test

## Discussion

The prevalence rates of *N. mikurensis* infection were 5% for both the PMR patients and the control subjects, which are a bit lower than the prevalence rates of 7.4% and 9% monitored in Norwegian patients treated with biological therapies and blood donors, respectively [17, 18], but identical to the prevalence rate of 5% among Swedish lymphoma patients [19].

The main findings of this study were that PMR patients infected with *N. mikurensis* differed from non-infected PMR patients by having higher levels of autoantibodies to vascular endothelium and an inflammatory profile characterized by elevated levels of the cytokine IL-1β and depressed levels of angiopoietin-1, cystatin C and IGFBP-4.

The pro-inflammatory IL-1β is released subsequent to triggering of the multiprotein complexes known as inflammasomes, which assemble in response to internal or external danger signals [20]. This system is activated in the defense against intracellular bacteria but also in autoimmune and auto-inflammatory disorders [20, 21] Despite its importance in infections and inflammatory conditions, it was unexpected to detect IL-1β in the blood of PMR patients as this cytokine is difficult to detect in patients on corticosteroid therapy [22].

Angiogenesis, i.e., the creation of new blood vessels from existing blood vessels by capillary sprouting, is regulated by a complex network of growth factors and cytokines that have stimulatory and inhibitory effects [23]. IGFBP-4 is one such inhibitory growth factor that negatively regulates angiogenesis mediated by the pro-angiogenetic growth factors insulin-like growth factor-1 and fibroblast-growth factor-2 [24]. Angiopoietins can assist in the remodeling of blood vessels by binding to the Tie 2 receptors on endothelial cells [23]. The reduced levels of IGFBP-4 and angiopoietin-1 in the serum of PMR patients infected with *N. mikurensis* might reflect a more pro-angiogenic environment in patients with an infection of the blood vessels. Cystatin C is a cysteine protease inhibitor found in all body fluids, which is clinically used as a biomarker for kidney function [25]. Reduced serum levels of cystatin C are seen in patients with inflamed and damaged arterial walls, e.g., atherosclerosis and aneurysms [26]. Moreover, bacterial danger signals such as lipopolysaccharide (LPS) and cytosine-phosphate-guanine oligodeoxynucleotides (CpG) reduce the release of cystatin C by immune cells [25]. Both mechanisms could explain the reduced serum cystatin C levels of PMR patients infected with *N. mikurensis*.

Anti-endothelial antibodies were one of the strongest discriminatory factors in the multivariate models for separation of the infected from the non-infected PMR patients. The moderately strong correlation found between IgM AECA and IgM to *N. mikurensis* implies that these antibody specificities may be interconnected – either through a causal relationship or cross-reactivity. Our previous observation that neoehrlichiosis patients with an intact immune system tend to develop arterial vascular events as opposed to the venous events seen in patients with compromised B-cell immunity implies that antibodies may be part of the pathology of the vascular events associated with *N. mikurensis* infection [3]. In a similar way, a recent study documented a strong link between the severity of COVID-19 and the occurrence of IgM autoantibodies with capacity to activate complement and negatively alter the function of vascular endothelium in vitro [27].

A limitation of this study is that it focused on PMR patients with active disease, most of whom had a low dose of oral corticosteroids, which is likely to have dampened and possibly modified the cytokine responses. In addition, the median duration of PMR in our cohort was 5 years. We cannot ascertain whether the *N. mikurensis* infection detected in 5% of the PMR patients was a recent event or not. The fact that *N. mikurensis* may be latent and asymptomatic,^4^ presumably for many years, makes it challenging to determine when the infection was contracted. We found identical rates of infection in the cohort of PMR patients and matched control subjects, which indicates that there is no accumulation of cases of *N. mikurensis* infection among patients with PMR in Sweden.

All 11 infected patients (8 with PMR and 3 controls) chose to treat the infection with antibiotics, but the study was not designed to evaluate the efficacy of eradication of the infection on PMR. We also considered whether any of the PMR patients with *N. mikurensis* infection might have been misdiagnosed as PMR. However, such a supposition would require a different study design, i.e., the systematic investigation of corticosteroid treatment-naïve patients provisionally diagnosed with PMR and evaluation if the “PMR” would be cured by eradication of *N. mikurensis* infection. We chose to interpret the data presented herein to show that the infected PMR patients had been correctly diagnosed with PMR, and that the superimposed infection with *N. mikurensis* had an impact on the inflammatory profile of these patients, which was skewed towards a more pro-inflammatory and pro-angiogenic profile. We are unable to ascertain if the *N. mikurensis* infection may have triggered PMR in any of these cases. However, the finding that infected patients had endothelial autoantibodies is intriguing and implies a possible pathogenic role of such autoantibodies in *N. mikurensis* infection that warrants further investigation.

## Supplementary Information

Below is the link to the electronic supplementary material.

Supplementary Material 1

## Data Availability

No datasets were generated or analysed during the current study.
