# Supplementary material for: The association of Neoehrlichia mikurensis infection with polymyalgia rheumatica
Source: Infection. 2026 Jul 10;54(5):2419–27. doi: 10.1007/s15010-026-02857-0 (PMC13727818; doi:10.1007/s15010-026-02857-0)
Supplement: Supplementary file 1 [file 15010_2026_2857_MOESM1_ESM.docx]

**Supplementary Material**

**Supplementary Fig. 1.** Geographic location of the primary care centers that recruited PMR patients to the study

The seven primary care clinics in the Västra Götaland Region of Sweden that recruited PMR patients were Närhälsan vårdcentral of Norrmalm, Billingen, Herrestad, Dagson, Sandared, Ågårdskogen and Bollebygd.

| **Symptoms** | **Co-morbidities** |
| --- | --- |
| Achilles tendon rupture | Diabetes |
| Arm pain | Heart disease |
| Back pain | Hematologic disorder |
| Bruising due to corticosteroid treatment | History of thrombosis |
| Chills | History of heart disease |
| Cold feet | Immunodeficiency |
| Constipation | Kidney disease |
| Cramps in calves and feet | Previously treated for cancer |
| Depression | Pulmonary disease |
| Difficulty initiating movement, slow motor function | Skin disease |
| Dizziness |  |
| Dry eyes |  |
| Easily stumbles, weakness |  |
| Eyes watering |  |
| Fatigue |  |
| Feeling unwell |  |
| Feeling warm (especially at night) |  |
| Fever |  |
| Fragile skin |  |
| Headache |  |
| High blood pressure |  |
| Lack of energy / fatigue |  |
| Leg cramps / Calf cramps during walking |  |
| Mental fatigue / brain fog |  |
| Morning stiffness |  |
| Movement pain |  |
| Muscle weakness / Weakness in arms, pelvis and legs |  |
| Nausea |  |
| Neck pain |  |
| Night sweats |  |
| Nocturnal pain |  |
| Numbness in legs and feet |  |
| Pain reduced with pain medication |  |
| Palpitations |  |
| Pelvic pain |  |
| Poor appetite |  |
| Rash |  |
| Restless legs |  |
| Severe cramps (legs, hands) |  |
| Shoulder pain |  |
| Stiffness |  |
| Sweating (mostly in the evening) |  |
| Swelling (feet, hands, eyelids, etc.) |  |
| Temporal arteritis |  |
| Thigh pain |  |
| Thin skin |  |
| Weakness (Fatigue due to all symptoms) |  |
| Worsened cataract |  |
|  |  |

**Supplementary Table 1.** Symptoms and co-morbidities of PMR patients

**Supplementary Table 2**. Inflammatory markers and antibodies analyzed in serum samples

| **Cytokines** | **Inflammatory profile**  **at diagnosis of PMR** | **Antibodies** |
| --- | --- | --- |
| Angiopoietin-1 | B-Hemoglobin | Cytosolic AECA IgG |
| Angiopoietin-2 | WBC | Cytosolic AECA IgM |
| CXCL8 - Cytokine C-X-C motif chemokine ligand 8 | B-Platelets | Membrane AECA IgG |
| Cystatin C | S-C-reactive protein | Membrane AECA IgM |
| EGF - Epidermal Growth Factor | S-Albumin | Borrelia IgG |
| FGF-basic - Fibroblast Growth Factor basic | S-Antitrypsin | Borrelia IgM |
| TGF-β - Transforming Growth Factor Beta | S-Haptoglobin | *N. mikurensis* IgG |
| GM-CSF - Granulocyte-Macrophage Colony-Stimulating Factor | S-IgA | *N. mikurensis* IgM |
| ICAM-1 - Intercellular Adhesion Molecule 1 | S-IgG |  |
| IFN-α2 - Interferon Alpha-2 | S-IgM |  |
| IFN-β - Interferon Beta | S-Orosomucoid |  |
| IFN-γ - Interferon Gamma | ESR |  |
| IFN-λ1 - Interferon Lambda 1 |  |  |
| IFN-λ2/3 - Interferon Lambda 2/3 |  |  |
| IGFBP-4 - Insulin-like Growth Factor Binding Protein 4 |  |  |
| IL-10 - Interleukin-10 |  |  |
| IL-12p70 - Interleukin-12 p70 |  |  |
| IL-17A - Interleukin-17A |  |  |
| IL-1β - Interleukin-1 Beta |  |  |
| IL-2 - Interleukin-2 |  |  |
| IL-4 - Interleukin-4 |  |  |
| IL-6 - Interleukin-6 |  |  |
| IL-8 - Interleukin-8 (also known as CXCL8) |  |  |
| IP-10 - Interferon gamma-induced protein 10 |  |  |
| MCP-1 - Monocyte Chemoattractant Protein-1 |  |  |
| MMP-2 - Matrix Metalloproteinase-2 |  |  |
| MMP-9 - Matrix Metalloproteinase-9 |  |  |
| MPO - Myeloperoxidase |  |  |
| MRP8/14 - Myeloid-related Protein 8/14 |  |  |
| Myoglobin |  |  |
| NGAL - Neutrophil Gelatinase-Associated Lipocalin |  |  |
| OPN - Osteopontin |  |  |
| PECAM-1 - Platelet Endothelial Cell Adhesion Molecule-1 |  |  |
| PlGF - Placental Growth Factor |  |  |
| SAA - Serum Amyloid A |  |  |
| TNF-α - Tumor Necrosis Factor Alpha |  |  |
| VCAM-1 - Vascular Cell Adhesion Molecule-1 |  |  |
| VEGF - Vascular Endothelial Growth Factor |  |  |
